# Mechanical Behavior and Damage Mode Identification of Wind Turbine Blade GFRP Shear Webs Based on Acoustic Emission Detection Technology

**DOI:** 10.3390/s26082363

**Published:** 2026-04-11

**Authors:** Luopeng Xu, Jiajun Zheng, Wenkai Wang, Zhixin Li, Huawei Zou

**Affiliations:** 1 School of Science, Civil Aviation Flight University of China, Guanghan 618307, China; lizhixin_com@126.com; 2 Sichuan Province Engineering Technology Research Center of General Aircraft Maintenance, Civil Aviation Flight University of China, Guanghan 618307, China; 3 State Key Laboratory of Advanced Polymer Materials, Polymer Research Institute, Sichuan University, Chengdu 610065, China; 4 Aviation Engineering Institute, Civil Aviation Flight University of China, Guanghan 618307, China; jiajunz2023@163.com (J.Z.); wwk971210@outlook.com (W.W.)

**Keywords:** wind turbine blade shear web, glass fiber-reinforced polymer (GFRP), acoustic emission (AE), hierarchical agglomerative clustering (HAC), K-means clustering, damage mode identification

## Abstract

This study investigates the acoustic emission (AE) response and damage mode characteristics of ±45° glass fiber-reinforced polymer (GFRP) composites used in wind turbine blade shear webs under quasi-static tensile loading. It aims to establish the relationship between AE features and three typical damage mechanisms—matrix cracking, interfacial debonding, and fiber fracture—to support damage assessment and structural health monitoring. Quasi-static uniaxial tensile tests with synchronous AE monitoring are conducted on specimens with three orientations (0°, 45°, and 90°). AE features are selected using correlation analysis and principal component analysis, and the HAC-initialized K-means clustering method is employed for damage mode identification. The optimal number of clusters is determined to be three, according to the Davies–Bouldin index (DBI) and the Silhouette index (SI). The resulting low-, mid-, and high-frequency clusters are associated with matrix cracking, interfacial debonding, and fiber fracture, respectively. These interpretations are further supported by wavelet-based time–frequency analysis and microscopic fracture surface observations.

## 1. Introduction

**Figure 1:**
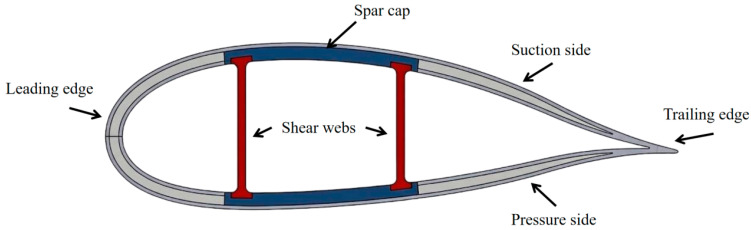
Wind turbine blade cross-section and shear web location.

With the advancement of global renewable energy strategies, wind power has become an important part of the clean energy system [1]. Meanwhile, the structural reliability of wind turbine units during long-term service has become a key factor influencing the sustainable development of the industry. Due to their high specific strength, high specific modulus, and excellent design flexibility, fiber-reinforced composites are widely used in large wind turbine blades, and they play an important role in the lightweight and large-scale development of blade structures [2]. Among the main load-bearing components of a wind turbine blade, the shear web connects the upper and lower spar caps, improves the overall stiffness and stability of the box girder structure, and transfers shear loads (Figure 1). To take full advantage of their in-plane shear capacity, shear webs are typically fabricated from glass fiber-reinforced polymer (GFRP) laminates with a ±45° lay-up design [3]. However, under complex and variable service loads, damage in composite shear webs often initiates at the microscale and progressively evolves through matrix cracking, interfacial debonding, and fiber fracture, ultimately leading to structural failure [4]. Because this damage evolution is typically hidden and cumulative, conventional inspection methods often have difficulty achieving timely detection and early warning. Therefore, developing nondestructive testing methods capable of real-time damage monitoring and in-service condition assessment is of great importance for ensuring the long-term safe operation of wind turbine blades and improving the life-cycle management of wind farms [5].

Acoustic emission (AE) technology captures damage activity in real time by detecting transient elastic waves released during material loading and therefore provides an effective means for in situ monitoring and the evaluation of damage evolution in composite structures. AE-based damage mode identification depends strongly on the selection of characteristic parameters and on the method used to associate these parameters with specific failure mechanisms. In early studies, damage classification is often based on a limited number of AE indicators, such as amplitude, counts, energy, or frequency, together with empirically defined thresholds or decision boundaries. Panasiuk et al. [6] recorded multiple AE parameters during quasi-static tensile tests of polyester-based GFRP plates and used amplitude and hit count as the main criteria to distinguish matrix elastoplastic deformation, matrix cracking accompanied by fiber/matrix debonding, and fiber fracture. Santulli et al. [7] reanalyzed AE data from transverse tensile and single-fiber fracture tests of carbon/epoxy laminates and reported that matrix cracking-related signals were mainly concentrated around 100 kHz. These studies demonstrate that suitable AE parameters can provide a preliminary basis for distinguishing damage modes in composite materials. However, traditional parameter-based approaches usually rely on empirically selected thresholds and are sensitive to the material system, loading condition, and sensor layout. When large volumes of AE data are involved, it becomes difficult to achieve both objectivity and automation, which limits the applicability of such methods under complex service conditions [8].

With the development of machine learning and pattern recognition, increasing attention is given to data-driven approaches for AE-based damage mode identification. Zhou et al. [9] selected rise time, duration, energy, peak amplitude, and frequency as clustering features in uniaxial tensile tests of carbon fiber-woven composites. Using K-means clustering together with frequency range analysis and microscopic observations, they classified AE signals into matrix cracking, fiber/matrix debonding, and fiber fracture. Li et al. [10] performed the clustering analysis on AE signals generated during tensile loading of two-dimensional and three-dimensional woven glass/epoxy composites. Through the Laplacian score and correlation analysis, they identified peak amplitude and peak frequency as the most effective clustering features and further distinguished matrix cracking, delamination, and fiber bundle fracture using K-means++ combined with principal component analysis. Zhao et al. [11] combined AE monitoring with digital image correlation in tensile tests of carbon/glass hybrid woven composites and employed fuzzy C-means clustering to classify AE signals into matrix cracking, interfacial debonding, and fiber fracture.

Related studies further expand the use of clustering methods in composite damage analysis. Gulsen et al. [12] applied ensemble feature selection to improve AE-based damage mode identification in CFRP honeycomb sandwich structures and showed that counts, energy, and partial powers made strong contributions to mode discrimination. Zhang et al. [13] investigated GFRP main spar materials with prefabricated delamination and wrinkle defects and identified AE event clusters associated with different defect types through multiparameter clustering analysis. Their results revealed the AE characteristics and corresponding damage evolution mechanisms under different defect conditions. Overall, these studies indicate that AE analysis based on multiparameter features and unsupervised clustering can effectively extract latent damage information and improve the automatic identification of multiple damage mechanisms in composites. Nevertheless, most existing studies are conducted on standard laboratory specimens. In addition, the selection of cluster number and algorithm parameters still considerably depends on prior experience, and conventional K-means remains sensitive to the initial cluster centers, which may reduce clustering stability [14]. Systematic studies on AE clustering analysis and damage evolution identification for actual wind turbine blade shear webs made of ±45° GFRP, particularly under different specimen orientations relative to the marked reference line, are still limited.

To address these issues, this study focuses on ±45° glass fiber-reinforced polymer (GFRP) wind turbine blade shear webs. With the marked reference line on the shear web taken as the 0° reference direction, tensile specimens with three orientations (0°, 45°, and 90°) are prepared and tested under quasi-static uniaxial tension with synchronous AE monitoring. On this basis, a damage mode identification procedure based on AE characteristic parameters is established. Key AE features are selected through correlation analysis and principal component analysis to construct the clustering feature space. Hierarchical agglomerative clustering (HAC) is then introduced to optimize the initial cluster centers of K-means for unsupervised clustering of AE events. Finally, the clustering results are interpreted together with time–frequency characteristics and microscopic fracture surface observations to identify the typical microscale damage mechanisms in ±45° GFRP shear webs.

## 2. Materials and Methods

### 2.1. Specimen Preparation

**Figure 2:**
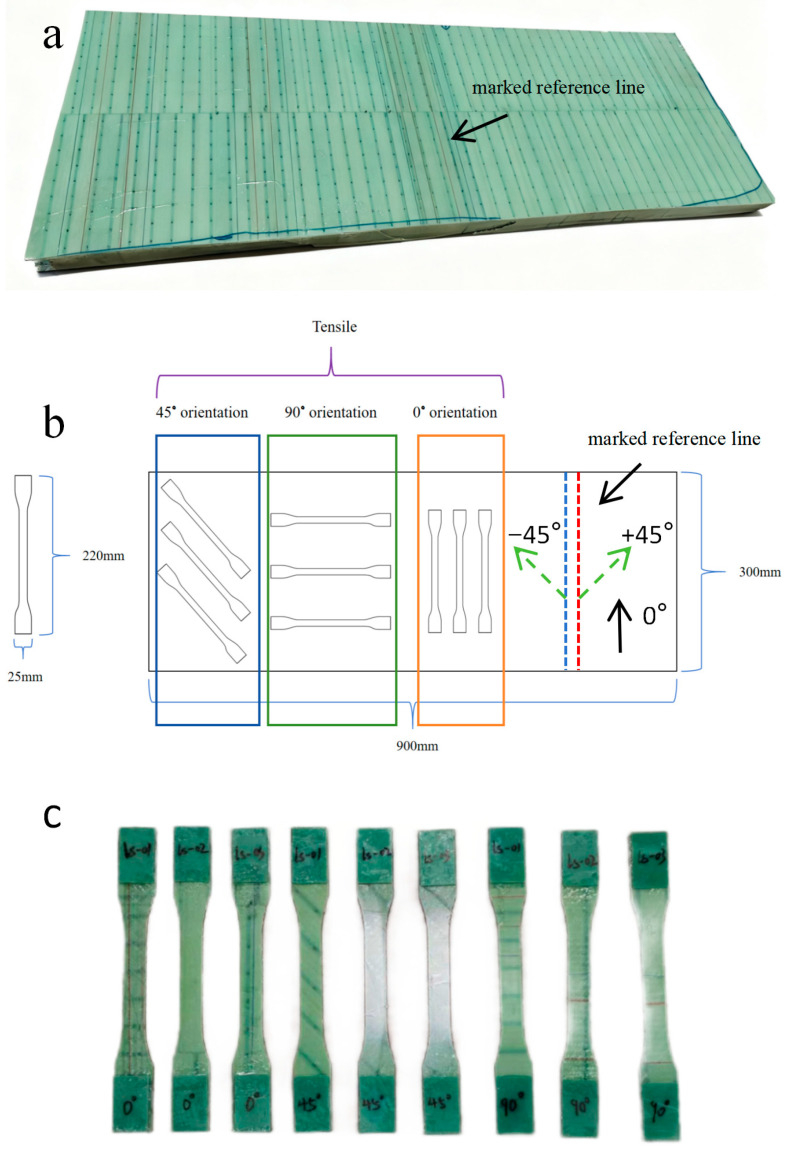
Specimen preparation procedure for the wind turbine blade shear web panel: (**a**) raw shear web panel; (**b**) schematic of 0°, 45°, and 90° specimen extraction and layout; (**c**) photograph of the specimens.

To investigate the damage evolution mechanisms of wind turbine blade composite materials, tensile specimens with different orientations are prepared from an actual wind turbine blade shear web panel following the procedure shown in Figure 2. The original shear web panel is shown in Figure 2a. The panel is manufactured through vacuum infusion and consists of four plies of ±45° biaxial glass fiber fabric. The lay-up sequence can be expressed as [(±45)]_4_, indicating that all plies adopt the same ±45° biaxial fabric configuration. The reinforcement is Taishan Fiberglass AX-808 biaxial glass fiber fabric with an areal weight of 808 g/m^2^ (Tai’an, China), and the matrix is an AT40 epoxy resin system supplied by Dongfang Dongshu New Materials (Mianyang, China). The material system, lay-up configuration, and manufacturing process are consistent with those of the actual shear web component.

**Figure 3:**
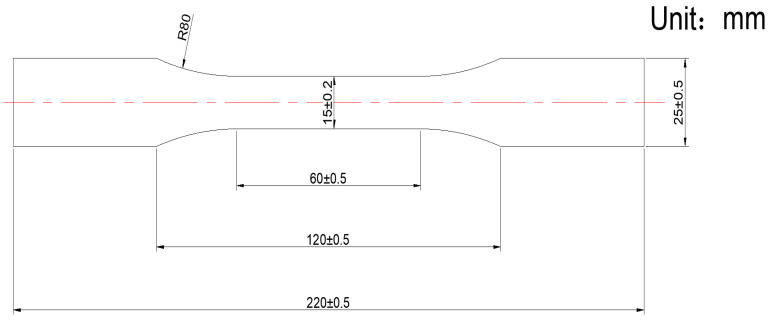
Dimensions of the tensile specimen.

During specimen preparation, the shear web panel is first separated from the original sandwich structure, and the foam core is removed to reduce the coupling effects of the sandwich interfaces and the through thickness structure on the overall mechanical response and acoustic emission (AE) wave propagation characteristics, thereby enabling the tests to focus more specifically on the damage response of the shear web panel material itself. During blade manufacturing, red and blue tracer lines are pre-marked on the panel surface along the blade longitudinal direction, and these tracer lines are used as the reference for defining the specimen orientations. Accordingly, the tracer line direction is defined as the 0° direction in this study. As shown in Figure 2b, since the shear web is fabricated from ±45° biaxial glass-fiber fabric, the two principal fiber bundle directions are oriented at +45° and −45° with respect to this reference line, as indicated by the green arrows. Based on this definition, the specimens cut parallel to the reference line are defined as 0° specimens, those cut parallel to one principal fiber bundle direction are defined as 45° specimens, and those cut perpendicular to the reference line are defined as 90° specimens. The sampling locations are selected in regions with smooth surfaces and with no obvious defects, and a low-heat-input cutting process is used to cut strips along the 0°, 45°, and 90° directions. Standard tensile specimens are then machined from these strips according to the geometric dimensions shown in Figure 3, with a specimen thickness of 2 mm. By designing the transition filets and the grip end dimensions, the fracture is ensured to occur preferentially within the effective gauge section. After machining, the specimen edges are deburred and lightly polished, and the gauge section is marked. To prevent premature failure or slippage at the grip ends during tensile loading, glass fiber-reinforced tabs are bonded to both ends of each specimen for end reinforcement, as shown in Figure 2c. After the tabs cure, the specimen ends are trimmed, and the flatness and key dimensions of the specimens are checked to ensure that all specimens meet the requirements for the subsequent tensile tests and synchronized AE monitoring.

### 2.2. Experimental Setup

**Figure 4:**
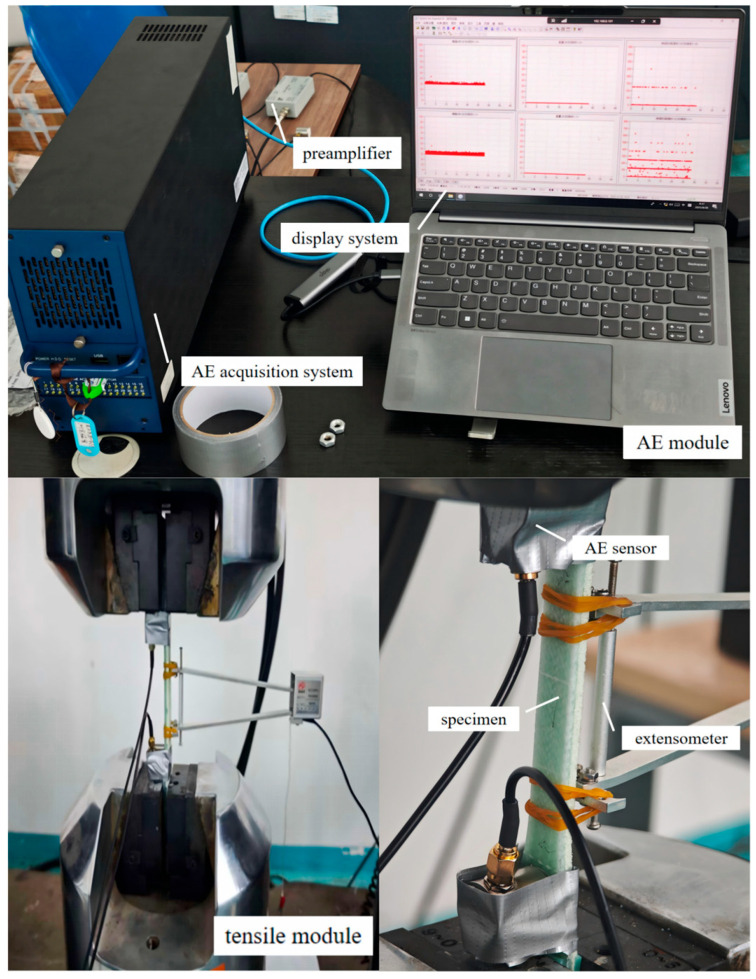
Tensile acoustic emission (AE) experimental monitoring platform.

The composite tensile tests are conducted in accordance with ASTM D3039 [15]. A synchronized mechanical loading and AE monitoring platform is established by our group (Figure 4). This platform consists of a tensile loading system and an AE data acquisition system, enabling synchronized recording of the mechanical response and AE signals during the tests.

The quasi-static tensile tests are performed using an ETM305D computer-controlled electronic universal testing machine (Shenzhen Wance, Shenzhen, China) with a maximum load capacity of 300 kN. The tests are conducted under displacement control at a speed of 1 mm/min, and all mechanical data are acquired and recorded in real time. AE signals are acquired using a PAC Micro-II Express multichannel system (Physical Acoustics Corporation, Princeton Junction, NJ, USA), with a PCI-2 high-speed acquisition card as the core acquisition hardware. Two R15α narrowband resonant sensors (center frequency: 150 kHz) and Model 2/4/6 preamplifiers (gain: 40 dB) are used. The sensors are mounted on the specimen surface using petroleum jelly as the couplant, and they are secured with cloth tape to ensure stable and reliable signal transmission.

Specimens with 0°, 45°, and 90° orientations are tested, with three replicates for each orientation. Two AE sensors are arranged along the specimen axis with a spacing of approximately 80 mm to synchronously acquire full-waveform data and AE characteristic parameters. The AE parameter settings are listed in Table 1. The detailed test procedure is as follows:

Step 1: Turn on the universal testing machine and the AE acquisition system; check that the power supply, signal cables, and preamplifier connections are functioning properly; and warm up the equipment for approximately 10 min.

Step 2: Mount the specimen in the grips of the testing machine and adjust the clamping position to ensure that the specimen axis is aligned with the loading direction. Tighten the grips to prevent slippage during loading.

Step 3: Install two R15α sensors at the designated positions on the specimen. Apply petroleum jelly as the couplant and secure the sensors with cloth tape, then connect the preamplifiers and acquisition channels. With no external load applied, operate the testing machine under no-load conditions to perform a background noise test and record the noise amplitude range. Based on the results, adjust the gain, filter bandwidth, and initial trigger threshold. Pencil lead break tests are then conducted to confirm the normal triggering of each channel and the absence of abnormal false triggering.

Step 4: Set the control mode of the testing machine to displacement control with a loading rate of 1 mm/min, and zero the load, displacement, and AE timing parameters to complete the preparations prior to the formal test.

Step 5: During the mechanical test, start AE acquisition and mechanical data recording, and then initiate tensile loading. Record the load and displacement, together with AE event parameters and full-waveform signals, in real time. Continue loading until a macroscopic fracture occurs while synchronously recording the specimen ID and macroscopic damage morphology.

Step 6: After the mechanical loading test, stop loading and data acquisition, save and back up all mechanical and AE data files, remove the specimen and sensors, clean the couplant from the specimen surface, and check the equipment status to prepare for the next test.

### 2.3. Attenuation Test

**Figure 5:**
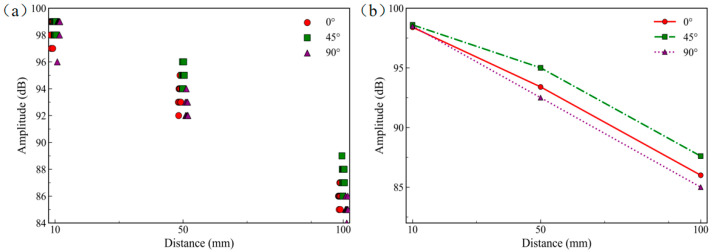
Pencil lead break (PLB) test results: (**a**) signal amplitude at different distances; (**b**) attenuation curves of the PLB signal for different orientations.

The Hsu–Nielsen pencil lead break (PLB) method is employed as a standard calibration procedure to verify the sensitivity of the AE sensors and to evaluate the signal attenuation characteristics in the composite specimen. In this method, a 0.5 mm lead is broken at an included angle of approximately 30° to simulate a standard AE source [16]. Along the specimen axis, PLB tests are performed at distances of 10 mm, 50 mm, and 100 mm from the sensor. For each specimen, three repeated PLB tests are conducted at each distance. Figure 5a presents the individual measurement points at different propagation distances. The results show that the signal amplitude generally decreases with increasing propagation distance. In addition, the amplitudes at all measurement locations remain higher than 80 dB, indicating that the sensors maintain high sensitivity over the entire test range. For each orientation, the three repeated measurements at the same propagation distance are first averaged for each specimen. The resulting mean values from the three parallel specimens are then further averaged to obtain the characteristic amplitudes listed in Table 2, which are used to plot the attenuation curves in Figure 5b.

As shown in Table 2 and Figure 5b, the AE amplitude in all three orientations decreases with increasing propagation distance from 10 mm to 100 mm. Specifically, at 10 mm, the amplitude ranges from 98.4 to 98.6 dB, whereas at 100 mm, it ranges from 85.0 to 87.6 dB. In terms of specimen orientation, the 45° orientation exhibits the smallest overall attenuation (11.0 dB) and shows a nonlinear attenuation trend: the amplitude reduction is relatively small over 10–50 mm, while the attenuation rate increases over 50–100 mm. For the 0° orientation, the amplitude decreases from 98.4 dB to 86.0 dB, corresponding to a total attenuation of 12.4 dB; the attenuation over the two distance intervals is relatively comparable, indicating a more uniform decreasing trend overall. For the 90° orientation, the amplitude decreases from 98.5 dB to 85.0 dB, giving the largest total attenuation (13.5 dB) among the three, and it also shows a relatively stable, near-linear attenuation trend. Overall, as the propagation distance increases, the amplitude differences among the three orientations slightly widen; however, the 45° orientation consistently maintains a higher amplitude and lower attenuation, indicating evident orientation-dependent wave propagation behavior in this ±45° shear web panel. This difference may be related to the anisotropic architecture of the material. In the 45° orientation, the wave propagation direction is closer to one fiber bundle direction, so the propagating wave is likely to cross fewer resin-rich regions and interfaces, which contributes to relatively weaker scattering, damping, and mode conversion, and thus lower attenuation. In contrast, in the 0° and 90° orientations, the larger angle between the propagation direction and the fiber bundles makes the wave interaction with the fiber bundles, resin matrix, and interlaminar interfaces more complex, which likely leads to greater energy loss during propagation.

## 3. Acoustic Emission Feature Parameters and Clustering-Based Identification Method

### 3.1. Acoustic Emission Feature Parameters and Data Preprocessing

**Figure 6:**
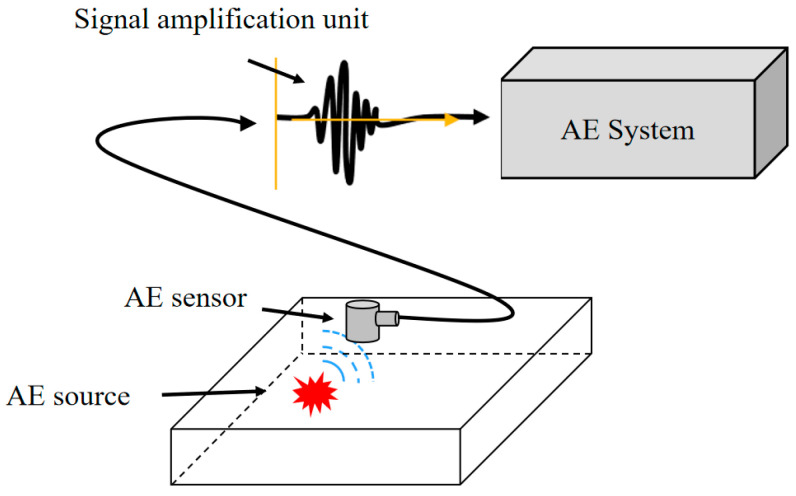
Acoustic emission testing system.

The AE system (Figure 6) consists of an AE source, an AE sensor, a signal amplification unit, and a data acquisition and processing unit [17]. When localized damage events such as microcrack initiation, fatigue crack growth, or frictional wear occur within the composite, high-frequency transient elastic waves are generated and propagated through the material. The AE sensor mounted on the specimen surface converts these waves into electrical signals, which are then amplified by the signal amplification unit (preamplifier) and subsequently transmitted to the AE system for data acquisition and processing.

**Figure 7:**
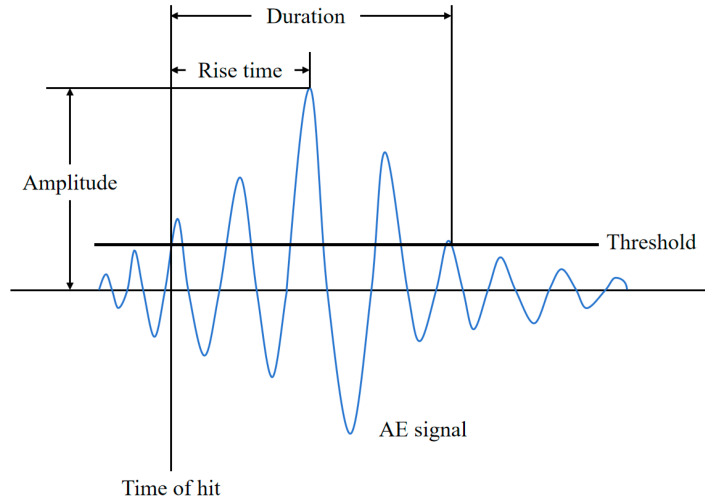
AE signal feature extraction.

Figure 7 illustrates the AE signal feature extraction process. By extracting features from the raw AE waveforms, multidimensional AE feature parameters can be obtained [18]. The time-domain features include amplitude (A), energy (E), counts (C), duration (D), RMS, ASL, and rise time (R), among others. The frequency-related features include average frequency (AF), reverberation frequency (RF), and initial frequency (IF), as well as centroid frequency (CF) and peak frequency (PF) in the spectrum. When the dataset contains too many feature dimensions, information overlap and redundancy are likely to occur, which not only increases the complexity of subsequent analysis but also weakens the stability and interpretability of the clustering results. Therefore, feature selection and dimensionality reduction are required to simplify the data structure while retaining key information, thereby obtaining a clearer and more effective signal representation.

Before AE feature selection, the raw AE hit data are preprocessed [19]. Spurious events unrelated to material damage are removed, including records with zero energy or amplitude, suspected noise points with extremely short duration and amplitude close to the triggering threshold, and a small number of saturated signals whose amplitudes far exceed the normal operating range of the system. Subsequently, Z-score standardization is applied to the feature data to eliminate the influence of differences in units and magnitudes among features on distance-based clustering results [20]. The standardization is expressed as follows:

(1) $$Z\;=\frac{x-\mu }{\sigma }$$

where *x* denotes the original value of a given feature, *μ* and *σ* denote the mean and standard deviation of that feature across all valid AE events, respectively, and *Z* denotes the standardized value. After standardization, each feature has a mean of 0 and a standard deviation of 1.

### 3.2. Feature Correlation Analysis and Principal Component Analysis (PCA) Method

After preprocessing and standardization of the AE feature data, it is still necessary to select a small number of features with low redundancy and higher sensitivity to damage evolution from the multidimensional feature set for subsequent clustering analysis. In this study, a Pearson correlation coefficient matrix is first used to identify highly correlated feature pairs with overlapping information. Principal component analysis (PCA) is then employed to evaluate the contribution of each feature to the overall variance and its loadings in the major principal components. Based on these results, the key AE feature parameters used for clustering are determined.

The Pearson correlation coefficient is used to quantify the linear correlation between any two AE features [21]. For the two feature variables, x and y, the Pearson correlation coefficient, r, can be expressed as:

(2) $$r\;=\frac{\sum_{i=1}^{n}\left(x_{i}-\overset{-}{x}\right)\left(y_{i}-\overset{-}{y}\right)}{\sqrt{\sum_{i=1}^{n}{\left(x_{i}-\overset{-}{x}\right)}^{2}}\sqrt{\sum_{i=1}^{n}{\left(y_{i}-\overset{-}{y}\right)}^{2}}}$$

where $x_{i}$ and $y_{i}$ are the feature values corresponding to the *i*-th AE event, $\overset{-}{x}$ and $\overset{-}{y}$ are the mean values of the corresponding features over all events, and n is the total number of valid events. Correlation coefficients are calculated for all selected features and are typically presented in a symmetric correlation matrix. The closer the absolute value of r is to 1, the stronger the correlation; values closer to 0 indicate a weaker correlation.

Meanwhile, PCA is introduced to perform the overall dimensionality reduction and structural analysis of the multidimensional features [22]. Based on the standardized feature matrix, a covariance matrix is constructed, and its eigenvalues and eigenvectors are computed. Let *λ_k_* denote the eigenvalue of the *k*-th principal component; its explained variance ratio can be expressed as:

(3) $$\eta_{k}\;=\;\frac{\lambda_{k}}{\sum \lambda_{k}}$$

The cumulative explained variance ratio is used to quantify how well the first *k* principal components explain the overall variability of the original data. During dimensionality reduction, principal components are selected in descending order of explained variance ratio to ensure that the main information in the original data is retained. On this basis, the contribution of each feature to the major principal components can be evaluated using the principal component loadings (i.e., the coefficients of the original features on each principal component, which reflect the strength of their association with the component). A larger absolute loading indicates that the feature contributes more significantly to the variation captured by the corresponding principal component. In this way, key features that contribute substantially to the major variation can be identified, while parameters with minor contributions or redundant information can be removed, thereby constructing a more compact and interpretable feature space for subsequent clustering analysis.

### 3.3. HAC–K-Means Hybrid Clustering Method

Conventional K-means clustering aims to minimize the within-cluster sum of squared errors (SSEs) by partitioning the samples into *K* clusters [23]. The objective function is given by:

(4) $$J\;=\sum_{j=1}^{K}\sum_{x_{i}\in C_{j}}{\left\|x_{i}-\mu_{j}\right\|}^{2}$$

where $x_{i}$ denotes the feature vector of the i-th sample, $C_{j}$ denotes the j-th cluster, and $\mu_{j}$ denotes the centroid of the j-th cluster.

*K*-means clustering is simple to implement and computationally efficient. However, the clustering results are sensitive to the initial centroids, and the number of clusters, *K*, must be specified in advance. To reduce the dependence on random initialization and improve the stability of the clustering results, a hybrid clustering procedure that combines HAC and *K*-means was adopted [24]. HAC employed a bottom-up agglomerative strategy, using Euclidean distance to measure sample similarity and the Ward minimum variance criterion to update inter-cluster distances, i.e., at each step merging the pair of clusters that yields the smallest increase in the within-cluster sum of squares [25]. Specifically, the standardized AE feature subset was used as input to HAC to obtain clustering partitions for different candidate numbers of clusters, and the corresponding Davies–Bouldin index (DBI) and Silhouette Index (SI) were calculated to comprehensively determine an appropriate number of clusters, *K* [26]. With the selected *K*, the centroids of each cluster were computed based on the HAC partition and used as the initial cluster centers for *K*-means. *K*-means was then run in the same feature space to iteratively optimize the clustering until the objective function converged.

### 3.4. Frequency-Domain Analysis of AE Waveforms and Multi-Scale Wavelet Analysis Method

AE signals are typical non-stationary transient signals. Their characterization requires not only attention to transient features in the time-domain waveform but also the examination of the energy distribution across different frequency bands. In this study, the fast Fourier transform (FFT) is used to obtain global frequency-domain characteristics, and the discrete wavelet transform (DWT) is applied to selected AE events for multi-scale analysis [27].

By applying FFT to the time-domain signal, $x\left(t\right)$, its frequency-domain representation can be obtained as:

(5) $$X\left(f\right)\;=\int_{-\infty }^{+\infty }x\left(t\right)e^{-j2\pi ft}dt$$

From the amplitude spectrum, $\left|X\left(f\right)\right|$, the dominant frequency, bandwidth, and energy distribution of the signal can be obtained. FFT is simple and intuitive; however, it provides only global spectral information and cannot reflect the local time evolution characteristics of the signal along the time axis.

To further characterize the local energy distribution of the signal across different frequency bands, the discrete wavelet transform (DWT) is introduced to perform multi-scale decomposition of the AE waveforms. Considering that AE signals are typically transient, impulsive, and non-stationary, the db4 wavelet is selected as the mother wavelet because of its good time–frequency localization capability for short-duration transient signal analysis and its wide use in related AE studies. An L-level decomposition is then applied to the discrete signal, yielding the detail components, d1–dL, and the approximation component, aL. With a fixed sampling frequency, each wavelet component can be approximately associated with a group of adjacent frequency sub-bands, thereby decomposing the original signal into local components within different frequency ranges [28]. In this study, the sampling frequency is 2 MHz. Therefore, a five-level decomposition is adopted as a compromise between frequency resolution and interpretability. If the decomposition level is lower, the resulting frequency bands are too broad to effectively distinguish the low-, mid-, and high-frequency components of the AE signals; if the decomposition level is higher, the frequency bands become overly fine and reduce the interpretability of the results. Under a five-level decomposition, the approximate frequency ranges corresponding to a5, and d5–d1 are 0–31.25, 31.25–62.5, 62.5–125, 125–250, 250–500, and 500–1000 kHz, respectively.

To quantitatively compare the energy allocation among different frequency bands, the energy of the *k*-th wavelet component (e.g., aL or dL) is defined based on its discrete wavelet coefficients $c_{k}\left(n\right)$ as:

(6) $$E_{k}\;=\sum_{n}{\left|c_{k}\left(n\right)\right|}^{2}$$

After normalization, the energy proportion can be obtained as:

(7) $$e_{k}\;=\frac{E_{k}}{\sum_{m}E_{m}}$$

where $\sum_{m}E_{m}$ denotes the sum of the energies of all wavelet components. By comparing the energy proportions in each frequency band among different events, the relative contributions of low-, mid-, and high-frequency components can be quantitatively identified, providing multi-scale frequency-domain support for distinguishing features associated with different damage modes.

## 4. Experimental Results

### 4.1. Analysis of Mechanical Properties

**Figure 8:**
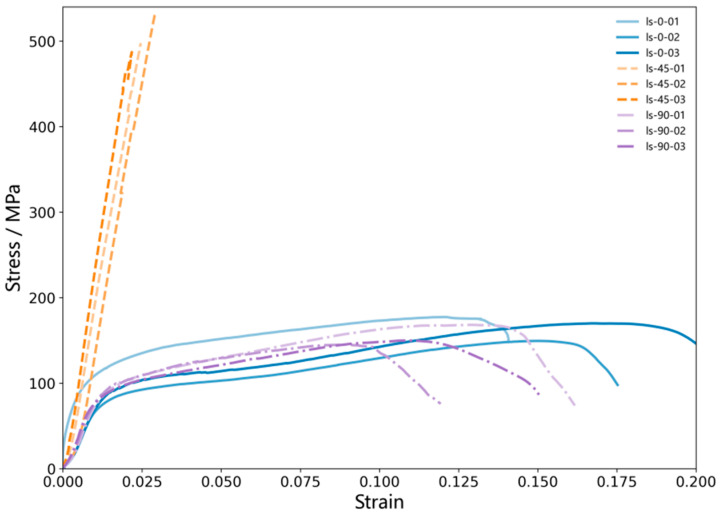
Stress–strain curves of the GFRP specimens with 0°, 45°, and 90° orientations.

Figure 8 shows the stress–strain curves of the ±45° GFRP composite specimens with 0°, 45°, and 90° orientations. The specimen ID follows the format “ls-angle-index”, where “ls” denotes the tensile test, the angle indicates the specimen orientation (0°, 45°, or 90°), and the index (01–03) denotes the replicate number for the same orientation. For example, ls-0-01 represents the first specimen with the 0° orientation. As can be seen, the 45° specimens exhibit an approximately linear response in the elastic stage, with the highest initial slope and the highest ultimate strength but a relatively small ultimate strain. After reaching the peak, the stress drops rapidly without an obvious nonlinear plateau, indicating rapid post-peak instability. The initial slope of the 0° specimens is lower than that of the 45° specimens and close to that of the 90° specimens. In the strain range of 0.01–0.03, the response gradually transitions from linear to nonlinear, followed by a prolonged gradual hardening–softening process. The ultimate strength is approximately 150–180 MPa, and the ultimate strain is about 0.15–0.18, with a relatively gradual post-peak stress reduction. It should be noted that the specimen ls-0-01 shows a slightly higher response in the initial small-strain stage than the other two 0° replicates. This difference is considered to mainly reflect the normal local variability of the composite specimens and slight fluctuations in the initial loading response, while the overall stress–strain evolution, strength level, and failure characteristics of the three 0° specimens remain generally consistent. The 90° specimens have an initial slope comparable to that of the 0° specimens, an ultimate strength of approximately 140–170 MPa, and an ultimate strain of about 0.10–0.15; additionally, they overall enter the softening/instability stage earlier.

The uniaxial quasi-static tensile test data of the GFRP specimens are summarized in Table 3. The ±45° GFRP composite exhibits pronounced anisotropy under different specimen orientations, which mainly arises from changes in the relative angle between the specimen axis and fiber bundles as the specimen orientation varies. This, in turn, alters the load transfer path and the dominant damage mechanisms. In terms of strength, the specimens with the 45° orientation show the highest average tensile strength of 505.2 MPa, whereas the average strengths for the 0° and 90° orientations are 165.5 MPa and 154.5 MPa, respectively. The average tensile strength of the 45° specimens is approximately 3.1 times that of the 0° and 90° specimens, indicating that the relative orientation between the fiber bundles and the loading axis has a significant influence on the load-carrying capacity of the material. Specifically, for the 45° orientation, one set of fiber bundles in the ±45° lay-up is approximately parallel to the specimen axis, such that the axial load can be more effectively transferred along the fiber direction and is primarily carried by the fibers, resulting in a higher tensile strength. In contrast, for the 0° and 90° orientations, the specimen axis is not parallel to either of the two fiber bundle directions in the lay-up; instead, both are oriented at ±45° relative to the axis. Therefore, the tensile load cannot be directly carried by the fibers and is more likely to be transferred through the resin matrix and the fiber/matrix interfaces, accompanied by progressive damage such as matrix shear cracking and interfacial debonding. Because the load-carrying capacity of the resin and interfaces is substantially lower than that of the fibers, the material strength is limited by these constituents, leading to failure at lower stress levels. In addition, the coefficients of variation for the 0°, 45°, and 90° orientations are 8.77%, 4.54%, and 7.87%, respectively, all below 10%, indicating good repeatability of the experimental data.

Overall, the 45° specimens exhibit higher strength and stiffness, and their failure is mainly characterized by fiber-dominated brittle fracture. In contrast, the 0° and 90° specimens show much lower strength but a more pronounced nonlinear plateau in the stress–strain curves, suggesting that their deformation and damage evolution are more strongly governed by matrix and interfacial shear behavior, resulting in more evident energy dissipation and progressive damage characteristics.

### 4.2. Coupling Between Mechanical Response and AE Parameters

The cumulative energy is defined as the sum of the energies of all AE events during the test, and it can effectively characterize the cumulative damage process and the energy release rate within the material. Analyzing the load–time curve alone only reflects the evolution of the specimen’s macroscopic load-carrying capacity over time and cannot adequately capture changes in internal damage activity. By comparing the cumulative energy curve with the mechanical load curve, the damage evolution of the composite during tensile loading can be more clearly revealed by jointly considering the mechanical response and energy release.

**Figure 9:**
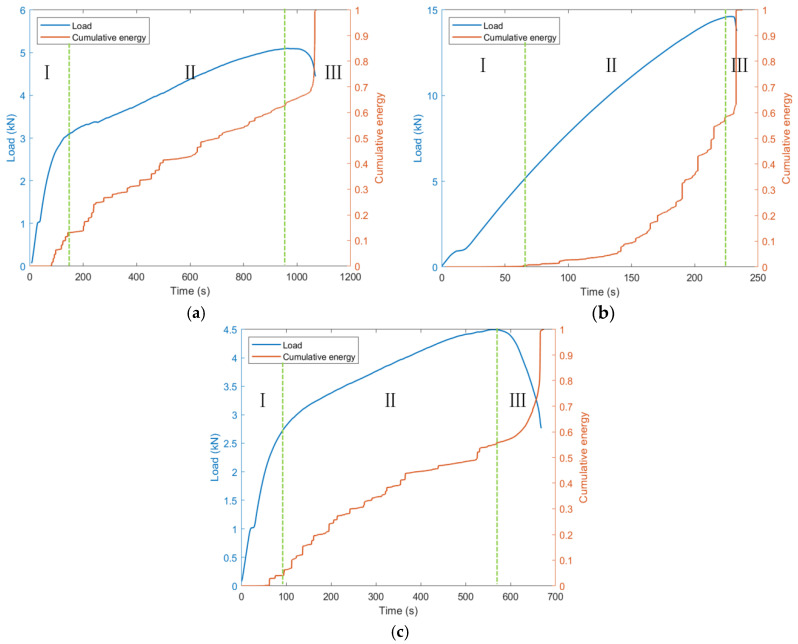
Cumulative AE energy versus time, together with the load–time curves, for specimens with different orientations. (**a**) 0° orientation (ls-0-03); (**b**) 45° orientation (ls-45-03); (**c**) 90° orientation (ls-90-03).

Figure 9 shows the load–time curves and the corresponding cumulative energy-time curves for specimens with 0°, 45°, and 90° orientations. The cumulative energy is normalized to vary from 0 to 1. Overall, based on changes in the growth rate of the normalized cumulative energy curve and in conjunction with key inflection points in the load curve, the loading process is divided into three stages (I–III), although the characteristics of each stage differ among the three orientations.

For the 0° orientation, during Stage I, the load increases approximately linearly, while the cumulative energy increases slowly, which may be associated with the initiation of matrix microcracks and early local interfacial debonding. In Stage II, the load continues to rise with a slightly reduced slope, and the cumulative energy shows a relatively steady increase, which may be related to the further propagation of matrix cracks and interfacial damage. After entering Stage III, the load decreases rapidly after reaching the peak, and the cumulative energy exhibits a pronounced jump within a short time, corresponding to macrocrack penetration and the failure of part of the load-bearing fiber bundles. For the 45° orientation, the cumulative energy remains at a relatively low level and varies smoothly in Stages I and II, suggesting that internal damage activity in the early stage is relatively weak. As the load approaches the peak and enters Stage III, the cumulative energy increases rapidly within a very short time, while the load drops sharply, which is inferred to be associated with sudden failure processes such as concentrated fiber bundle fracture. In contrast, for the 90° orientation, the cumulative energy increases more noticeably from Stage I, the growth interval in Stage II is relatively long, and a large increase is still maintained in Stage III, overall corresponding to the continuous development of matrix cracking and interfacial debonding from the early loading stage until final failure.

**Figure 10:**
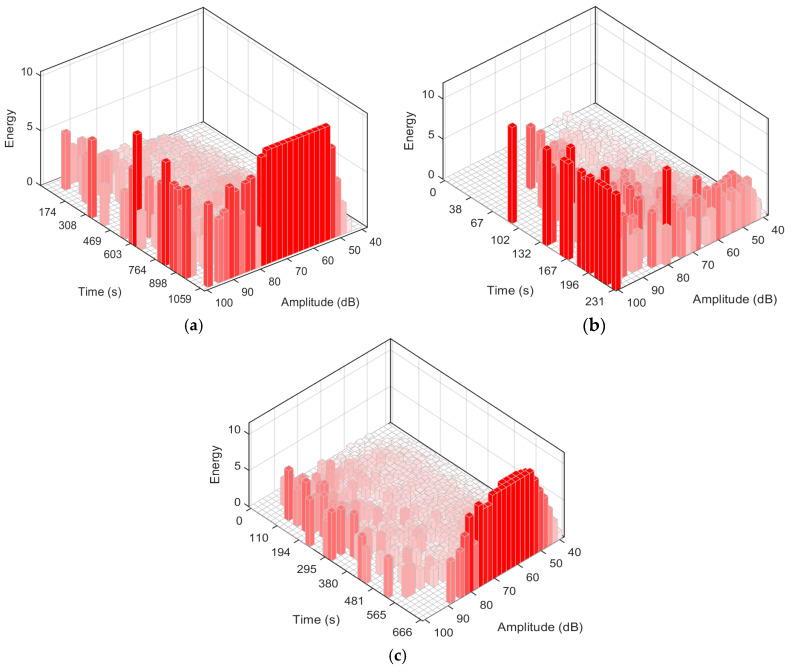
Three-dimensional plot of energy–time–amplitude for specimens with different orientations. (**a**) 0° orientation (ls-0-03); (**b**) 45° orientation (ls-45-03); (**c**) 90° orientation (ls-90-03).

Figure 10 shows the distribution of AE events in the energy–time–amplitude three-dimensional space for specimens with 0°, 45°, and 90° orientations. The time axis represents the measurement time from the start of loading to specimen failure. The amplitude axis represents the event peak amplitude, and the energy axis represents the cumulative AE energy within a given time window and amplitude interval, which is used to reflect the damage release intensity. Clear differences in the amplitude level and occurrence period of high-energy events can be observed among different orientations. For the 0° orientation, high-energy events are mainly concentrated in the moderate-amplitude region, with only a few events located in the high-amplitude region. These events occur mostly in the middle-to-late stage of loading, showing a trend in which moderate-amplitude, high-energy events gradually increase as loading proceeds. This phenomenon may be related to matrix cracking and interfacial debonding. For the 45° orientation, high-energy events also occur mainly in the middle-to-late stage, but their amplitude distribution evolves with time. In the early stage, high-energy events are dominated by the moderate-amplitude region. As the load approaches the peak and enters the failure stage, high-energy events gradually cluster in the high-amplitude region. This transition from the moderate-amplitude region to the high-amplitude region may indicate that the dominant damage mechanism shifts from matrix cracking and interfacial debonding to an increased contribution of the fiber fracture. For the 90° orientation, high-energy events are distributed across the moderate-to-high-amplitude range and persist from the middle stage to the late stage with a relatively dispersed distribution. This indicates that moderate-amplitude and high-amplitude energy events continue to occur over a wide time range. This behavior may be associated with the continuous accumulation of matrix cracking and interfacial debonding throughout loading, together with local fiber fracture contributing to failure.

## 5. Analysis and Discussion

In GFRP composites, establishing the correspondence between different damage modes and AE feature parameters is crucial for effective damage monitoring and identification. Previous studies have indicated that, compared with conventional amplitude-related parameters such as amplitude and energy [29], peak frequency shows higher sensitivity in distinguishing different microscopic damage mechanisms. To identify the dominant damage modes, an unsupervised clustering approach is adopted to automatically classify unlabeled AE signals.

### 5.1. AE Feature Selection and Dimensionality Reduction Analysis

**Figure 11:**
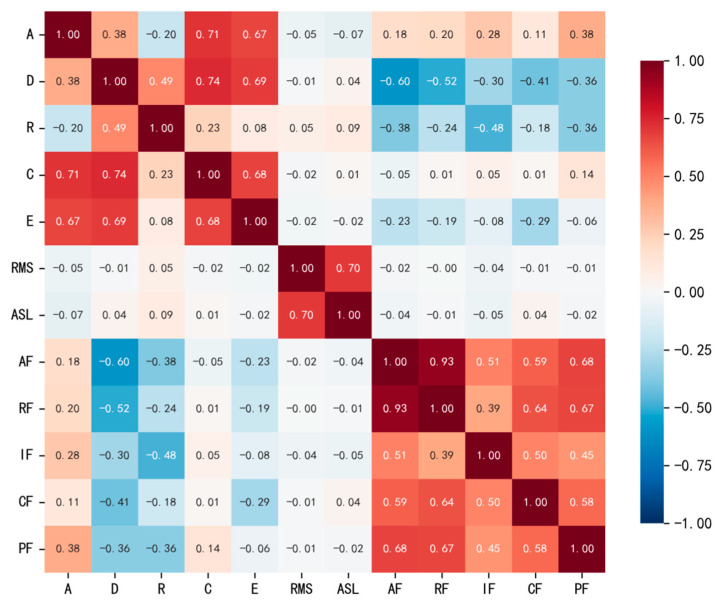
Pearson correlation coefficient matrix of AE feature parameters.

The Pearson correlation coefficient is mainly used to quantify the linear relationship between two AE features, while the correlation matrix reflects the overall correlation structure among multiple features. Figure 11 presents the Pearson correlation coefficient matrix of the AE features. As shown in the figure, the correlation coefficient between amplitude (A) and counts (C) is 0.71, that between counts (C) and duration (D) is 0.74, that between duration (D) and energy (E) is 0.69, that between amplitude (A) and energy (E) is 0.67, and that between counts (C) and energy (E) is 0.68, indicating relatively strong correlations among these parameters. Considering their physical definitions, these features may contain partially overlapping information in characterizing the response intensity, temporal persistence, and event scale of AE signals. Among the frequency-related parameters, the correlation coefficient between average frequency (AF) and reverberation frequency (RF) reaches 0.93, suggesting strong consistency between the two in describing spectral position. By contrast, peak frequency (PF) shows relatively weak correlations with the time-domain features, while the absolute correlation coefficients between rise time (R) and most other parameters generally remain below 0.5, indicating a relatively good degree of independence. In addition, the correlation coefficient between RMS and ASL is 0.70, and both parameters are more suitable for characterizing the level of continuous AE activity. Therefore, they are not taken as the primary input features for damage mode identification in this study. Taking into account the correlation relationships among parameters, their redundancy, and their physical meanings, amplitude (A), duration (D), rise time (R), and peak frequency (PF) are retained as candidate input features. Specifically, amplitude (A) is used to characterize the response intensity of AE events, duration (D) reflects the temporal persistence of the event, rise time (R) describes the signal onset behavior, and peak frequency (PF) provides relatively independent frequency-domain information. In comparison, the remaining parameters, which are either highly correlated or functionally similar, are not retained at this stage in order to reduce the dimensionality of the feature space and limit redundant information.

To further reduce the influence of feature redundancy on subsequent analysis, PCA is applied to the standardized candidate AE features to identify the major information dimensions and to support representative feature selection. PCA linearly transforms the potentially correlated original features into a set of uncorrelated principal components, which are ranked in descending order according to explained variance. On this basis, the retained principal components are determined according to their explained variance, and the contribution of each original feature is further analyzed using the corresponding loading coefficients. Representative AE parameters are then selected for subsequent clustering and damage identification.

**Figure 12:**
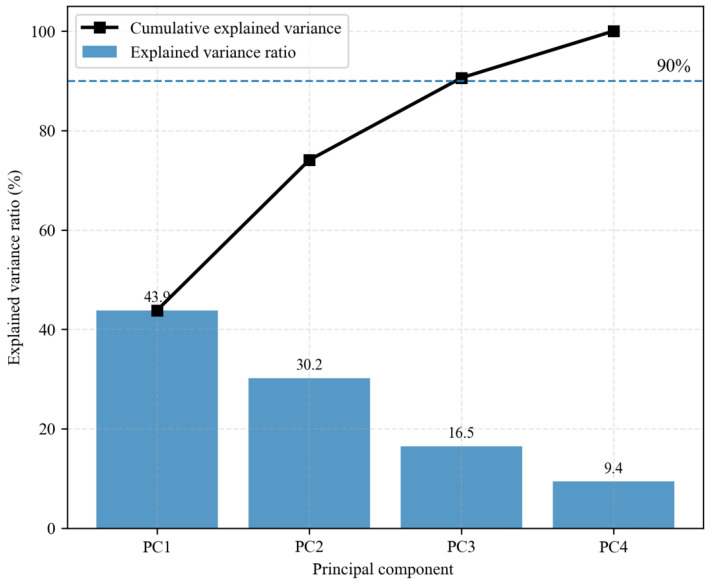
Explained variance and cumulative explained variance of the first four principal components.

Figure 12 presents the explained variance ratios and cumulative explained variance of the first four principal components. The explained variance ratios of PC1, PC2, PC3, and PC4 are 43.9%, 30.2%, 16.5%, and 9.4%, respectively. The cumulative explained variance of the first three principal components reaches 90.6%, indicating that most of the information in the original features is effectively captured by PC1–PC3. Accordingly, PC1–PC3 are regarded as the dominant information dimensions, and the contributions of the original features are further examined using the loading matrix.

Table 4 lists the loading coefficients of the standardized AE features on the first four principal components (PC1–PC4), which are used to interpret the major information dimensions captured by each component and to support feature selection. A larger absolute loading indicates a greater contribution of the corresponding feature to a given principal component, whereas the sign indicates only the direction of the relationship. As shown in Table 4, amplitude (A) has the largest absolute loading on PC2 (0.73), indicating that PC2 is closely associated with signal intensity. Rise time (R) has the largest absolute loading on PC3 (0.71), suggesting that PC3 mainly reflects the transient onset characteristics of the AE signal. Peak frequency (PF) shows relatively high loadings on both PC1 and PC3 (−0.58 and 0.65, respectively), indicating a substantial contribution to frequency-related variation. Taken together, A, R, and PF represent the major intensity, time-domain, and frequency-domain information in the AE dataset. In contrast, duration (D) also shows relatively high loadings on PC2 and PC4 (0.63 and 0.60, respectively), indicating its physical relevance to the temporal persistence of AE activity. However, D does not exhibit as distinct an independent role in the major principal components as A, R, and PF. Instead, its additional contribution is mainly reflected in PC4, which explains only 9.4% of the total variance. Therefore, although D retains physical relevance, its additional contribution to the overall variation structure is relatively limited, and it is not retained as an input feature for subsequent clustering.

Based on the feature selection results, the final AE feature subset consists of amplitude, peak frequency, and rise time. It should be noted that peak frequency shows relatively weak correlations with most time-domain features in the correlation analysis and exhibits high loadings on both PC1 and PC3 in PCA, further confirming its important role in distinguishing damage modes.

### 5.2. HAC–K-Means Hybrid Clustering

After feature selection, amplitude, rise time, and peak frequency are standardized, and the three-dimensional feature vector composed of these parameters is used as the input for clustering. HAC–K-means hybrid clustering is then performed on the AE events of specimens with 0°, 45°, and 90° orientations. First, HAC is applied in the standardized three-dimensional feature space defined by amplitude, rise time, and peak frequency, using Euclidean distance to measure the distance between samples, while the Ward method (minimum variance method) is used for cluster merging. Clustering is performed for different values of K in the range of 2–6, and the corresponding DBI (Davies–Bouldin index) and SI (Silhouette index) are calculated to evaluate the compactness within clusters and the separation between clusters. Then, for the selected K, the cluster centroids are calculated based on the HAC results and used as the initial cluster centers for K-means. K-means is subsequently run in the same feature space and iteratively updated until convergence to obtain stable clustering results.

**Figure 13:**
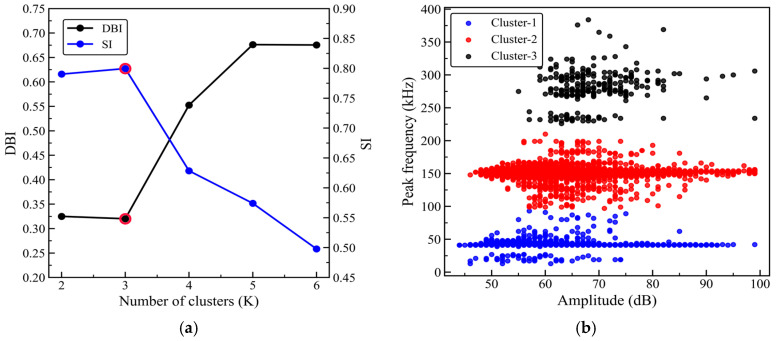
HAC–K-means clustering results for the 0° orientation. (**a**) Clustering evaluation metrics; (**b**) Clustering results.

**Figure 14:**
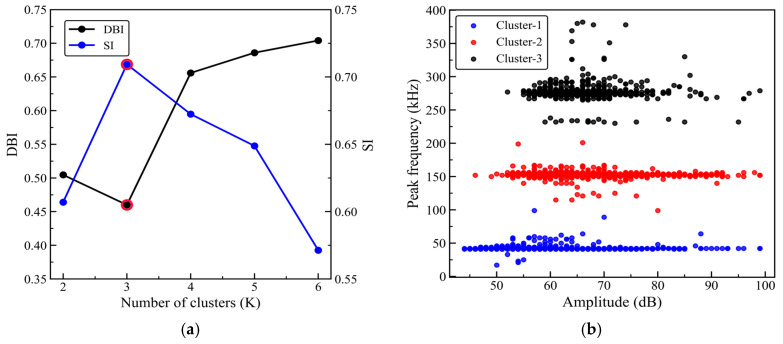
HAC–K-means clustering results for the 45° orientation. (**a**) Clustering evaluation metrics; (**b**) Clustering results.

**Figure 15:**
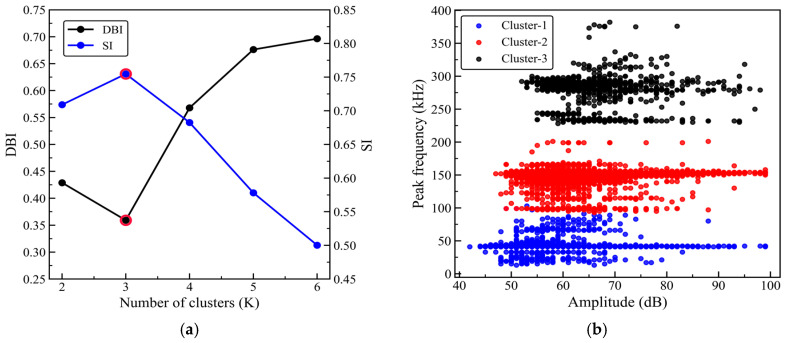
HAC–K-means clustering results for the 90° orientation. (**a**) Clustering evaluation metrics; (**b**) Clustering results.

Figure 13a, Figure 14a and Figure 15a show the DBI and SI curves as a function of the number of clusters K for the 0°, 45°, and 90° orientations. As K increases from 2 to 6, the DBI for all three orientations reaches its minimum at K = 3, while the SI reaches its maximum at K = 3. According to the criterion that a smaller DBI and a larger SI indicate better clustering performance, K = 3 can be considered to provide a reasonable balance between within-cluster compactness and between-cluster separation. Therefore, the number of clusters is consistently set to K = 3, and HAC–K-means clustering is then performed for each orientation; the results are shown in Figure 13b, Figure 14b, and Figure 15b, respectively. For subsequent comparative analysis, the three clusters are uniformly labeled as Cluster 1, Cluster 2, and Cluster 3, and the clustering results are projected onto the amplitude–peak frequency plane for visualization.

As shown in Figure 13b, Figure 14b and Figure 15b, although the distribution patterns of the point clouds and the cluster boundaries differ across the 0°, 45°, and 90° orientations, the three event types show relatively consistent band-like distributions in the peak frequency dimension. Overall, Cluster 1 is primarily located in the lower peak frequency region, with event amplitudes generally in the mid-to-low range; Cluster 2 is concentrated in the mid-frequency range, with relatively centered amplitude levels; and Cluster 3 is clearly skewed toward the higher peak frequency region, with a relatively higher lower bound of amplitude, indicating that these events have greater intensity levels.

To further assess the stability and repeatability of the HAC–K-means method, a repeated-run analysis is performed under the same clustering procedure. Specifically, 2000 AE events are randomly selected from each of the 0°, 45°, and 90° datasets to construct fixed subsets, and clustering is repeated 50 times on the same subsets. Conventional K-means adopts random initialization, whereas HAC–K-means determines the initial cluster centers based on the results of Ward’s linkage hierarchical clustering. Clustering performance is evaluated using the Davies–Bouldin index (DBI), Silhouette index (SI), and adjusted Rand index (ARI), as shown in Table 5. The results show that HAC–K-means consistently yields lower DBI values and higher SI values than conventional K-means for all three orientations, indicating improved intra-cluster compactness and inter-cluster separation. Meanwhile, the ARI values of conventional K-means are 0.720, 0.759, and 0.893 for the 0°, 45°, and 90° orientations, respectively, suggesting that the clustering results are still influenced by random initialization. In contrast, the ARI values of HAC–K-means are all 1.000, and the standard deviations of all evaluation metrics are close to zero, indicating highly consistent clustering results under fixed data and parameter settings. These results demonstrate that using HAC to initialize K-means effectively reduces the uncertainty associated with random initial centers, thereby improving the stability and repeatability of the clustering analysis.

Table 6 summarizes the feature statistics of the three clusters for the 0°, 45°, and 90° orientations, where the ranges are reported for amplitude and peak frequency, and the mean is reported for rise time. Cluster 1 corresponds to low-frequency events for all three orientations, with peak-frequency ranges of 13–93 kHz (0°), 17–99 kHz (45°), and 13–103 kHz (90°). The amplitude ranges are 44–99 dB, 47–99 dB, and 42–99 dB, respectively, with relatively low lower bounds, indicating that the events are more concentrated in the mid-to-low amplitude range. The mean rise times are 97.30 μs, 91.73 μs, and 104.98 μs, suggesting a relatively gentle signal onset and a slower initiation process for this cluster. Cluster 2 is mainly distributed in the mid-frequency range, with peak-frequency ranges of 97–210 kHz (0°), 99–201 kHz (45°), and 93–201 kHz (90°). The amplitude ranges are 46–99 dB, 46–99 dB, and 47–99 dB, respectively. The mean rise times are 43.66 μs, 33.43 μs, and 48.12 μs, which are markedly shorter than those of Cluster 1, indicating a steeper transient onset and faster initiation for this cluster. Cluster 3 corresponds to high-frequency events, with peak-frequency ranges of 226–384 kHz (0°), 230–382 kHz (45°), and 228–382 kHz (90°). The amplitude ranges are 55–99 dB, 52–99 dB, and 52–98 dB, respectively, with lower bounds higher than those of the first two clusters. The mean rise times are 25.68 μs, 32.64 μs, and 27.47 μs, which are the shortest among the three clusters, indicating a more rapid signal onset and a stronger transient release for this cluster.

Table 7 summarizes the characteristic frequency ranges of typical damage modes in GFRP composites reported in previous studies. In general, matrix cracking is mainly associated with the low-frequency range, interfacial debonding with the mid-frequency range, and fiber fracture with the high-frequency range. According to the peak-frequency distributions of the three clusters shown in Table 6, Cluster 1 falls within 13–93 kHz for 0°, 17–99 kHz for 45°, and 13–103 kHz for 90°, which are predominantly located in the low-frequency region commonly related to matrix cracking. Cluster 2 spans 97–210 kHz for 0°, 99–201 kHz for 45°, and 93–201 kHz for 90°, corresponding mainly to the mid-frequency range that is often associated with interfacial debonding, although some overlap with ranges reported in the literature should be acknowledged. Cluster 3 covers 226–384 kHz for 0°, 230–382 kHz for 45°, and 228–382 kHz for 90°, which are primarily distributed in the high-frequency range. Moreover, Cluster 3 exhibits relatively higher lower-bound amplitudes and shorter mean rise times, suggesting a more pronounced transient release behavior.

By combining the frequency ranges reported in the literature (Table 7) with the statistical characteristics of the present clusters (Table 6), Cluster 1, Cluster 2, and Cluster 3 can be reasonably interpreted as damage activities dominated mainly by matrix cracking, interfacial debonding, and fiber fracture, respectively. These results provide a useful qualitative basis for identifying the dominant damage characteristics of AE events from the unsupervised clustering results, although some degree of overlap and uncertainty among different damage mechanisms remains unavoidable.

**Figure 16:**
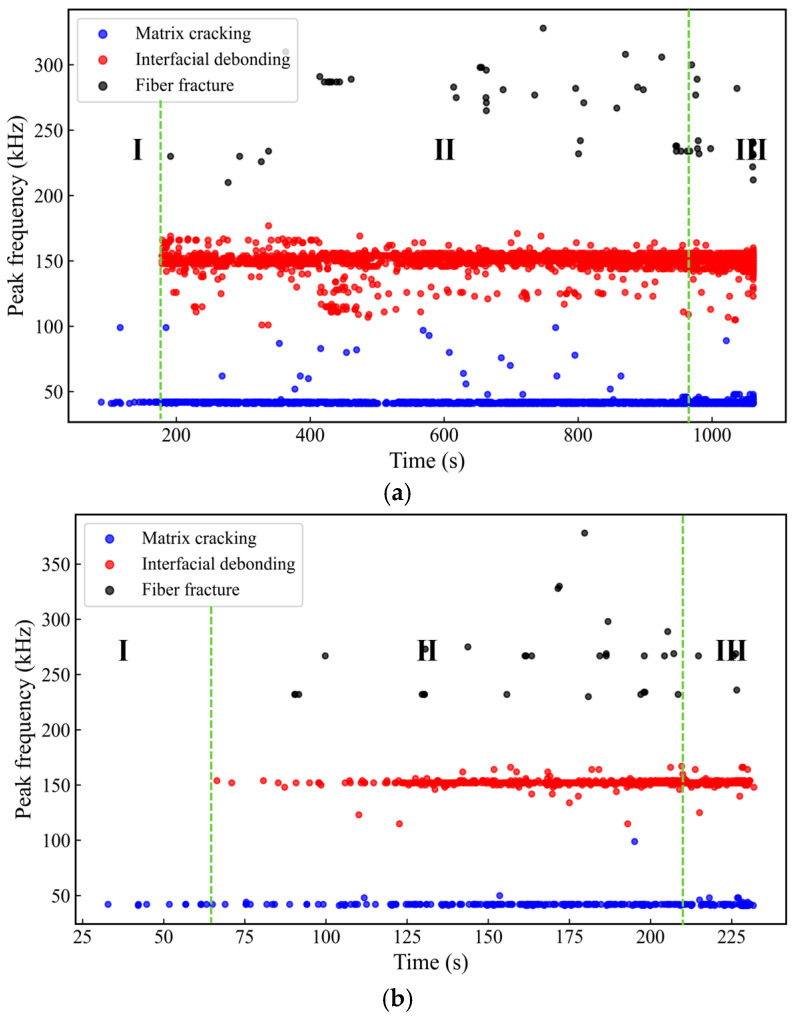

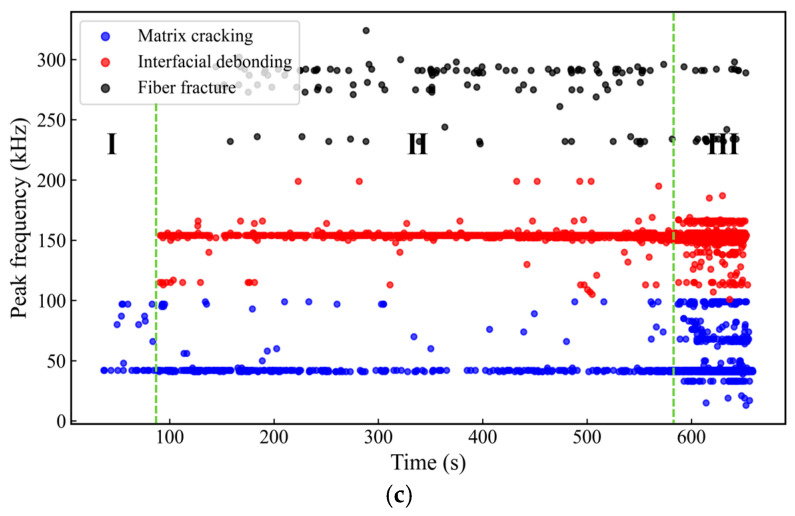
Time–peak frequency distribution of AE events in different stages for specimens with different orientations. (**a**) 0° orientation (ls-0-03); (**b**) 45° orientation (ls-45-03); (**c**) 90° orientation (ls-90-03).

Figure 16 shows the time–peak frequency distribution of AE events for specimens with 0°, 45°, and 90° orientations. Based on the load–time response, the loading process is divided into three stages (I–III). According to the clustering results and the frequency-band ranges reported in the literature, the three event types are attributed to matrix cracking, interfacial debonding, and fiber fracture. Overall, in the early stage of loading, matrix-cracking-, and interfacial debonding-related events dominate for all three orientations. As loading proceeds, the 0° and 90° orientations still mainly exhibit continuous accumulation of matrix and interfacial damage, whereas in the 45° orientation, fiber fracture events increase markedly after Stage II and persist into Stage III, indicating a transition of the dominant damage mode from matrix/interfacial-related damage to fiber fracture-dominated damage. Specifically, for the 0° orientation, relatively few events occur in Stage I. In Stage II, interfacial debonding events increase significantly and form a relatively continuous band-like distribution around 150 kHz, while high-frequency fiber fracture events appear only sporadically. In Stage III, as failure approaches, the numbers of all three event types increase, but matrix and interfacial damage remain dominant overall. For the 45° orientation, a large number of fiber fracture events appear from Stage II onward and form a more concentrated distribution around 250–300 kHz, indicating that fiber-related damage is more active in the middle-to-late stages and plays a more pronounced role in the final failure. In contrast, the evolution for the 90° orientation is similar to that for the 0° orientation. In Stage II, interfacial debonding events continue to dominate, and high-frequency events are limited in number and relatively dispersed. After entering Stage III, the event density of matrix and interfacial damage further increases, and, although high-frequency events increase to some extent, they do not become dominant.

### 5.3. AE Waveform and Spectral Characteristics

To better understand the differences among the three damage modes and to illustrate the variation in AE responses under different damage modes, representative events recorded during damage monitoring are analyzed from both the time-domain waveforms and the frequency-domain characteristics. Specifically, in the normalized feature space used for clustering, the Euclidean distance between each AE event and the corresponding cluster centroid is calculated, and the 10 events with the smallest centroid distances in each cluster are selected as candidate representative signals. Based on the waveform completeness and the time–frequency response characteristics of these candidate events, one typical AE event is finally selected from each cluster to represent matrix cracking, interfacial debonding, and fiber fracture, respectively. On this basis, FFT is applied to the corresponding raw signals to obtain their spectral distributions. By comparing the time-domain waveforms and the frequency bands in which the spectral energy is mainly concentrated for the three types of events, the characteristics of different damage modes can be identified more clearly.

**Figure 17:**
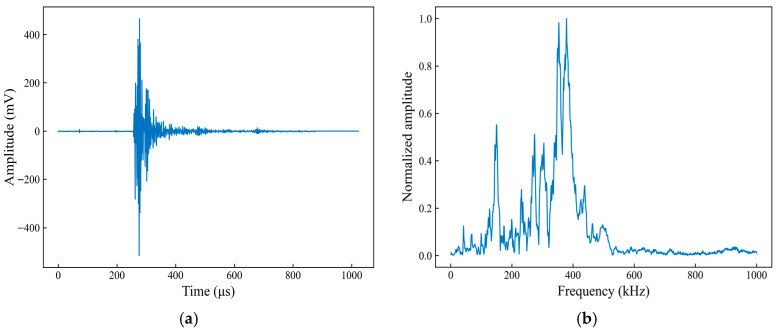
Time-domain waveform and spectral characteristics of a typical acoustic emission signal associated with fiber fracture. (**a**) Time-domain waveform; (**b**) Spectral characteristics.

Figure 17 shows the time-domain waveform and spectral characteristics of a typical fiber fracture AE signal. From the time-domain waveform (Figure 17a), the signal can be seen to rise rapidly at the onset, with the amplitude reaching a high level within a very short time. This is followed by dense high-frequency oscillations, and the ringing amplitude decays rapidly. The entire signal returns close to zero within a few hundred microseconds, exhibiting a short duration and pronounced transient characteristics. In the spectral characteristics plot (Figure 17b), the signal energy is primarily concentrated in the mid-to-high frequency range, with the main peak located in the 300–400 kHz range. Above 500 kHz, the amplitude decreases significantly, leaving only a few discrete components. The overall spectral distribution is relatively concentrated, highlighting the high-frequency nature of the fiber fracture events.

**Figure 18:**
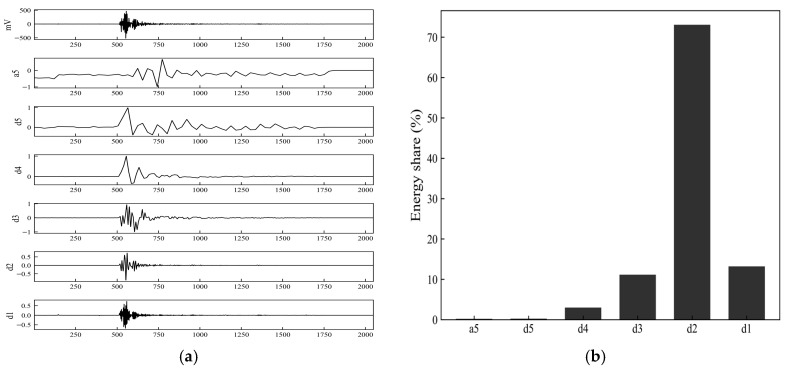
Wavelet decomposition coefficients and energy proportion in each sub-band for the acoustic emission signal associated with fiber fracture. (**a**) Wavelet decomposition coefficients; (**b**) Energy proportion in each sub-band.

To investigate the distribution of the fiber fracture AE signal across different frequency bands, wavelet transform is employed to perform multi-scale decomposition of the time-domain signal. The db4 wavelet, which is commonly used for analyzing transient and impulsive signals, is selected as the mother wavelet. A five-level discrete wavelet decomposition is applied to the time-domain signal, yielding the low-frequency approximation component, a5, and the detail components, d5–d1, from low to high frequency. The energy of each sub-band is calculated as the sum of the squares of the wavelet coefficients and is normalized to obtain the energy proportion in each sub-band. The results are shown in Figure 18.

Figure 18 shows the original signal and the wavelet coefficients of each sub-band, from top to bottom: the original waveform, the approximation component, a5, and the detail components, d5–d1. From the wavelet decomposition coefficients shown in Figure 18a, the amplitude of the original signal changes significantly during the main ringing phase, and notable spike features appear in the higher-frequency detail components, d2 and d1. Meanwhile, the coefficients of a5 and the lower-frequency sub-bands, such as d5 and d4, have smaller amplitude values and exhibit relatively smooth variations. Quantitative analysis of the energy distribution across the sub-bands, as shown in Figure 18b, indicates that the energy proportion of the d2 sub-band is 72%, followed by d3 and d1, whereas the remaining sub-bands account for relatively small proportions. This indicates that the energy of the fiber fracture event is primarily concentrated in the mid-to-high frequency scales corresponding to the d2 sub-band, with the high-frequency components of the signal being more prominent.

**Figure 19:**
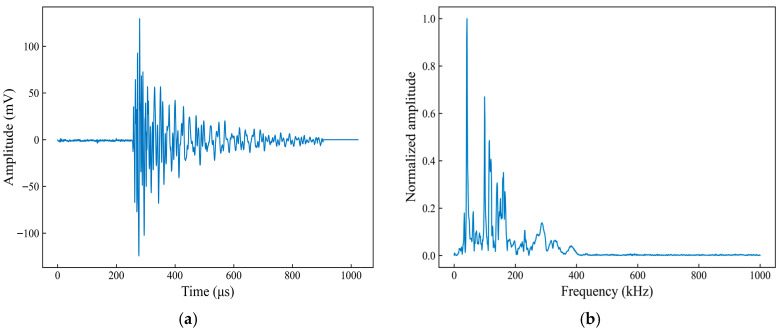
Time-domain waveform and spectral characteristics of a typical acoustic emission signal associated with matrix cracking. (**a**) Time-domain waveform; (**b**) Spectral characteristics.

Figure 19 shows the waveform and spectral characteristics of a typical AE signal associated with the matrix-cracking damage mode. From the time-domain waveform (Figure 19a), the signal onset occurs at 260.5 μs and reaches the maximum amplitude within a short time, followed by a sustained ringing stage, in which the amplitude gradually decays and returns to the baseline at 905.0 μs. Overall, this signal exhibits a relatively long duration and a smooth oscillation decay. From the frequency spectrum (Figure 19b), the signal energy is mainly distributed in the 0–200 kHz band, with a dominant peak in the low-frequency range and several secondary peaks within 100–200 kHz, whereas the amplitude above 300 kHz is low, and the high-frequency components are relatively limited. Taken together, these time- and frequency-domain characteristics indicate a long-ringing, low-frequency-dominated response, which can be regarded as a representative signal of the matrix-cracking damage mode.

**Figure 20:**
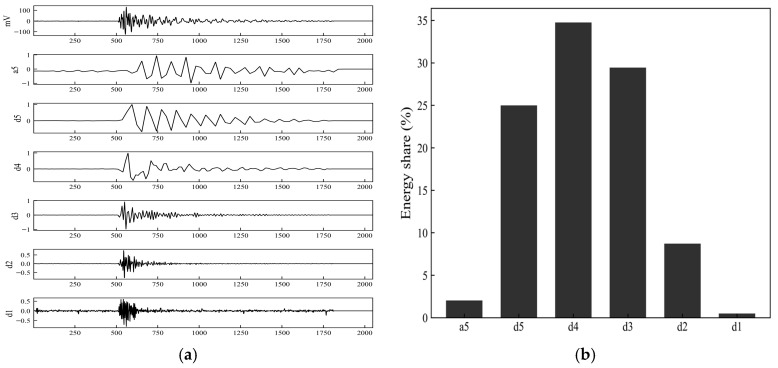
Wavelet decomposition coefficients and energy proportion in each sub-band for the acoustic emission signal associated with matrix cracking. (**a**) Wavelet decomposition coefficients; (**b**) Energy proportion in each sub-band.

Figure 20 presents the wavelet decomposition results and the energy proportions of each sub-band for a typical matrix-cracking AE signal. From the wavelet decomposition coefficients shown in Figure 20a, the main ringing of the original signal is more pronounced in the mid-to-low frequency detail components, d5, d4, and d3. Among them, the d4 sub-band shows relatively higher coefficient amplitudes and a longer duration, followed by d5 and d3, whereas the high-frequency detail components d2 and d1 exhibit much smaller amplitudes and only weak fluctuations at the onset of the signal. The energy proportion results shown in Figure 20b indicate that the energy proportions of the d4 and d3 sub-bands are 35% and 30%, respectively, and that d5 also accounts for a certain proportion, while the energy proportions of a5, d2, and d1 are all low. Overall, the energy of the matrix-cracking signal is mainly concentrated in the mid-to-low frequency sub-bands, with weak high-frequency components, reflecting a time–frequency characteristic dominated by mid-to-low frequency responses.

**Figure 21:**
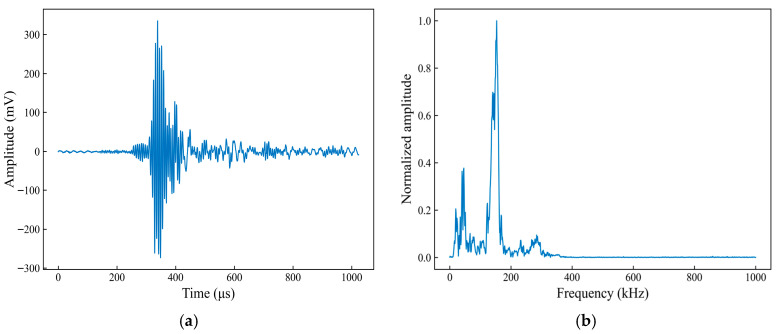
Time-domain waveform and spectral characteristics of a typical acoustic emission signal associated with interfacial debonding. (**a**) Time-domain waveform; (**b**) Spectral characteristics.

Figure 21 shows the waveform and spectral characteristics of a typical AE signal associated with the interfacial debonding damage mode. From the time-domain waveform (Figure 21a), the signal onset occurs at 258.5 μs and reaches the peak within a short time, followed by an extended ringing stage in which the amplitude gradually decreases to near zero at 1000 μs. Overall, the signal exhibits a relatively high amplitude and a more complex oscillation process in the time domain. From the frequency spectrum (Figure 21b), the signal energy is mainly distributed in the mid-frequency range of 100–200 kHz, with a dominant peak around 150–180 kHz and several secondary peaks in the lower-frequency range, while the amplitude above 300 kHz is close to zero and the high-frequency components are relatively limited. Taken together, the interfacial debonding signal shows amplitude and duration characteristics intermediate between those of matrix cracking and fiber fracture, and its spectrum is dominated by mid-frequency components, making it a representative response of the interfacial debonding damage mode.

**Figure 22:**
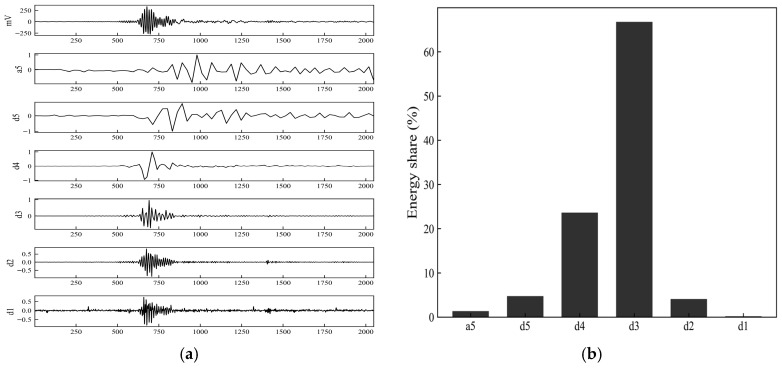
Wavelet decomposition coefficients and energy proportion in each sub-band for the acoustic emission signal associated with interfacial debonding. (**a**) Wavelet decomposition coefficients; (**b**) Energy proportion in each sub-band.

Figure 22 shows the wavelet decomposition results and the energy proportions of each sub-band for a typical interfacial debonding AE signal. From the wavelet decomposition coefficients shown in Figure 22a, the main ringing is most prominent in the d3 sub-band, where the coefficient amplitude is relatively high and the duration is longer. The d4 sub-band follows, with clear fluctuations observed during both the signal onset and decay phases, whereas the coefficients of a5, d5, and the high-frequency sub-bands d2 and d1 are relatively small. The energy proportion statistics shown in Figure 22b indicate that the d3 sub-band accounts for 65% of the energy, followed by the d4 sub-band with 25%, while the remaining sub-bands account for relatively small proportions. Overall, the energy of the interfacial debonding AE signal is primarily concentrated in the mid-frequency d3 sub-band, with some distribution in the adjacent d4 sub-band, reflecting a time–frequency characteristic dominated by mid-frequency components.

Based on the above analysis, the time–frequency characteristics of the typical signals and the results of the wavelet energy distribution analysis indicate that the three signal types obtained from clustering differ in their energy distribution characteristics. The waveform characteristics of the low-frequency, mid-frequency, and high-frequency clusters are consistent in terms of their overall trends, with the typical response characteristics of matrix cracking, interfacial debonding, and fiber fracture, respectively. Notably, the energy distribution of the wavelet sub-bands is well-aligned with the frequency-band characteristics of the cluster centers. These results support the qualitative damage mode identification based on parameter clustering, as discussed earlier, and improve the reliability of this method for distinguishing different damage mechanisms.

### 5.4. Microscopic Observation of Fracture Surfaces

**Figure 23:**
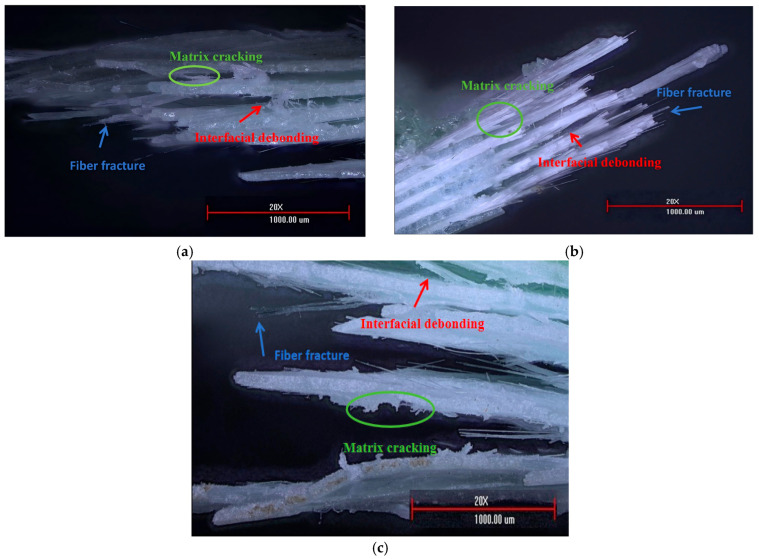
Microstructural images under different damage modes. (**a**) 0° orientation; (**b**) 45° orientation; (**c**) 90° orientation.

The fracture surfaces are examined using a PZ-CS350M 3D ultra-depth-of-field optical microscope (Beijing Pinzhichuangsi Precision Instruments Co., Ltd., Beijing, China). The system provides clear fracture morphology images over a large depth of field, enabling comparative observation of damage features in fiber-bundle regions, resin-rich regions, and interfacial areas. For each specimen orientation, multiple regions of the fracture surface are observed at different local positions and magnifications. Figure 23 presents representative microscopic images selected from these observations, with typical damage features annotated.

As shown in Figure 23, three typical damage morphologies—matrix cracking, interfacial debonding, and fiber fracture—are observed in specimens of all orientations, suggesting that failure generally involves the interaction of multiple damage mechanisms. Matrix cracking is mainly found in resin-rich regions (green-circled areas), interfacial debonding tends to develop along the fiber-matrix interface or interlaminar interfaces (red-arrowed areas), and fiber fracture is primarily concentrated in load-bearing fiber bundles (blue-arrowed areas).

The fracture morphology also varies with specimen orientation. The 45° specimens show more pronounced fiber fracture and fiber pull-out, indicating a stronger tendency toward fiber-dominated failure. In contrast, the 0° and 90° specimens exhibit more evident cracks in resin-rich regions and more obvious interfacial separation, reflecting the progressive accumulation of matrix cracking and interfacial debonding. The low-frequency AE cluster is generally associated with matrix-cracking features, the mid-frequency cluster appears to be more closely related to interfacial debonding, and the high-frequency cluster shows an overall correspondence with fiber fracture features. Accordingly, the relatively rapid increase in cumulative AE energy and the greater proportion of high-frequency events near peak load in the 45° specimens are consistent, at the overall trend level, with the more pronounced fiber fracture and pull-out observed on the fracture surfaces. By comparison, the earlier and more sustained increase in cumulative AE energy in the 0° and 90° specimens is generally consistent with the more evident matrix cracking and interfacial debonding features.

## 6. Conclusions

This study investigates a ±45° glass fiber-reinforced polymer (GFRP) wind turbine blade shear web panel. The specimens with 0°, 45°, and 90° orientations are prepared, and quasi-static tensile tests with synchronized acoustic emission (AE) monitoring are conducted. By integrating correlation analysis, principal component analysis (PCA), and the HAC-initialized K-means hybrid clustering method, the mechanical responses, AE evolution characteristics, and damage modes of specimens with different orientations are systematically analyzed. The clustering results are further examined using representative waveforms, frequency spectra, and microscopic fracture surface morphologies. The main conclusions are as follows:

(1) The 45° specimens exhibit the highest tensile strength, reaching 505.2 MPa, and they show a rapid loss of load-bearing capacity after the peak load, suggesting a fiber-dominated failure characteristic. In contrast, the 0° and 90° specimens show much lower tensile strengths of 165.5 MPa and 154.5 MPa, respectively. These two orientations also exhibit more pronounced pre-peak nonlinearity, indicating a more progressive damage accumulation process that is mainly associated with matrix- and interface-related deformation and damage.
(2) Based on the selected AE features of amplitude, rise time, and peak frequency, the HAC-initialized K-means clustering results indicate that the optimal number of clusters is three, with the DBI reaching its minimum and the SI reaching its maximum at K = 3. The AE events are thus divided into low-, mid-, and high-frequency clusters, with peak-frequency ranges of 13–103 kHz, 93–210 kHz, and 226–384 kHz, respectively. These three frequency ranges are mainly associated with AE events dominated by matrix cracking, interfacial debonding, and fiber fracture, thereby providing a useful frequency band-based reference for damage mode identification in ±45° GFRP shear webs. It should also be noted that this correspondence is established mainly from the clustering results and qualitative interpretation, and some overlap may still exist among the frequency ranges of different damage mechanisms.
(3) The db4 wavelet energy proportion analysis shows that fiber fracture-type events are mainly dominated by the mid-to-high frequency sub-bands, with the d2 sub-band accounting for 72% of the energy. Matrix cracking-type events are mainly concentrated in the mid-to-low frequency sub-bands, where the energy proportions of d4 and d3 are 35% and 30%, respectively. interfacial debonding-type events are mainly distributed in the mid-frequency sub-bands, with d3 and d4 accounting for 65% and 25% of the energy, respectively. These wavelet energy distribution results are generally consistent with the low-, mid-, and high-frequency classifications of the three event clusters. Microscopic fracture surface observations further reveal characteristic features such as matrix cracks, interfacial separation or debonding, and fiber fracture or pull-out, which further support the correspondence between the low-frequency cluster and matrix cracking, the mid-frequency cluster and interfacial debonding, and the high-frequency cluster and fiber fracture.

## Figures and Tables

**Table 1:** Main AE parameter settings.

Parameter	Value
Threshold	40 dB
Preamplifier gain	40 dB
Analog filter bandwidth	20 kHz–1 MHz
HDT (hit definition time)	200 μs
HLT (hit lockout time)	300 μs
PDT (peak definition time)	50 μs
Sampling rate	2 MHz
Sampling length	2048

**Table 2:** Attenuation signal data (dB).

	Distance	10 mm	50 mm	100 mm
Orientation				
0°		98.4	93.4	86.0
45°		98.6	95.0	87.6
90°		98.5	92.5	85.0

**Table 3:** Uniaxial quasi-static tensile test data of the GFRP specimens.

Specimen ID	Ultimate Load (kN)	Ultimate Strength (MPa)	Average Tensile Strength (MPa)	Coefficient of Variation
ls-0-01	5.32	177.3	165.5	8.77%
ls-0-02	4.48	149.3		
ls-0-03	5.10	170.0		
ls-45-01	14.93	497.7	505.2	4.54%
ls-45-02	15.93	531.0		
ls-45-03	14.61	487.0		
ls-90-01	5.05	168.3	154.5	7.87%
ls-90-02	4.36	145.3		
ls-90-03	4.50	150.0		

**Table 4:** Loading coefficient matrix for the first four principal components.

AE Parameters	PC1	PC2	PC3	PC4
Amplitude	−0.35	0.73	−0.15	−0.56
Rise time	0.57	0.15	0.71	−0.36
Duration	0.45	0.63	−0.19	0.60
Peak frequency	−0.58	0.20	0.65	0.44

**Table 5:** Stability comparison between conventional K-means and HAC–K-means.

Orientation	Method	*n*	DBI	SI	ARI
0°	K-means	2000	0.761 ± 0.193	0.557 ± 0.129	0.720 ± 0.242
0°	HAC–K-means	2000	0.302 ± 0.000	0.800 ± 0.000	1.000 ± 0.000
45°	K-means	2000	0.553 ± 0.173	0.659 ± 0.086	0.759 ± 0.298
45°	HAC–K-means	2000	0.462 ± 0.000	0.708 ± 0.000	1.000 ± 0.000
90°	K-means	2000	0.459 ± 0.150	0.715 ± 0.069	0.893 ± 0.108
90°	HAC–K-means	2000	0.350 ± 0.000	0.763 ± 0.000	1.000 ± 0.000

**Table 6:** Distribution of feature parameters for clusters in each specimen orientation.

	0° orientation		
	Amplitude/dB	Peak frequency/kHz	Rise time/μs
Cluster 1	44–99	13–93	97.30
Cluster 2	46–99	97–210	43.66
Cluster 3	55–99	226–384	25.68
	45° orientation		
	Amplitude/dB	Peak frequency/kHz	Rise time/μs
Cluster 1	47–99	17–99	91.73
Cluster 2	46–99	99–201	33.43
Cluster 3	52–99	230–382	32.64
	90° orientation		
	Amplitude/dB	Peak frequency/kHz	Rise time/μs
Cluster 1	42–99	13–103	104.98
Cluster 2	47–99	93–201	48.12
Cluster 3	52–98	228–382	27.47

**Table 7:** Characteristic frequency ranges of typical damage modes in GFRP composites.

Matrix Cracking	Interfacial Debonding	Fiber Fracture	References
10–150	150–250	350–500	Huguet et al. [30].
62.5–125	125–187.5	187.5–250	Beheshtizadeh et al. [31].
<60	200–320	380–430	Zhou et al. [9].
97–194	119–234	380–500	Palacios et al. [32].

## Data Availability

The data that support the findings of this study are available on request from the corresponding author. The data are not publicly available due to privacy or ethical restrictions.
